# A Constrained ICA-EMD Model for Group Level fMRI Analysis

**DOI:** 10.3389/fnins.2020.00221

**Published:** 2020-04-15

**Authors:** Simon Wein, Ana M. Tomé, Markus Goldhacker, Mark W. Greenlee, Elmar W. Lang

**Affiliations:** ^1^CIML, Biophysics, University of Regensburg, Regensburg, Germany; ^2^Experimental Psychology, University of Regensburg, Regensburg, Germany; ^3^IEETA/DETI, Universidade de Aveiro, Aveiro, Portugal

**Keywords:** independent component analysis, ICA, empirical mode decomposition, EMD, Green's-function - based EMD, fMRI

## Abstract

Independent component analysis (ICA), being a data-driven method, has been shown to be a powerful tool for functional magnetic resonance imaging (fMRI) data analysis. One drawback of this multivariate approach is that it is not, in general, compatible with the analysis of group data. Various techniques have been proposed to overcome this limitation of ICA. In this paper, a novel ICA-based workflow for extracting resting-state networks from fMRI group studies is proposed. An empirical mode decomposition (EMD) is used, in a data-driven manner, to generate reference signals that can be incorporated into a constrained version of ICA (cICA), thereby eliminating the inherent ambiguities of ICA. The results of the proposed workflow are then compared to those obtained by a widely used group ICA approach for fMRI analysis. In this study, we demonstrate that intrinsic modes, extracted by EMD, are suitable to serve as references for cICA. This approach yields typical resting-state patterns that are consistent over subjects. By introducing these reference signals into the ICA, our processing pipeline yields comparable activity patterns across subjects in a mathematically transparent manner. Our approach provides a user-friendly tool to adjust the trade-off between a high similarity across subjects and preserving individual subject features of the independent components.

## 1. Introduction

Independent component analysis (ICA) is a data-driven tool that is widely employed for functional magnetic resonance imaging (fMRI) data analysis. Based on a linear mixture model, either spatially (McKeown et al., 1998) or temporally (Biswal and Ulmer, 1999) independent components (ICs) can be obtained with ICA, without the requirement of prior information about anatomical regions of interest or temporal activation profiles. One problem of ICA is that, because of inherent indeterminacies, in general, it is not suitable for group studies. Different subjects have different time courses and spatial maps, and the extracted components will be sorted differently. This can make it difficult to find comparable activation patterns between subjects and draw inferences from subject groups. So far, various approaches have been proposed to overcome these shortcomings of ICA (Calhoun et al., 2009). Combining components obtained by single-subject ICA based on spatial correlation or clustering was proposed by Calhoun et al. (2001a) and Esposito et al. (2005). Another possibility is the spatial or temporal concatenation of the individual datasets to obtain components in a single ICA step from a group dataset, as well as the employment of back-reconstruction approaches to obtain subject-specific components (Calhoun et al., 2001b; Svensén et al., 2002). These concatenation-based approaches were compared with a simple across-subject averaging by Schmithorst and Holland (2004). In a sophisticated approach Beckmann and Smith (2005) proposed a tensorial extension of ICA. The authors extended the probabilistic ICA (PICA) model by adding a third dimension representing subject-related dependencies in addition to the spatio-temporal dimensions. The model represents a three-way factor analysis similar to the well-known PARAFAC model (Harshman and Lundy, 1994).

A popular paradigm used to acquire data for the above-mentioned exploratory matrix factorization techniques is the so-called resting-state. Under this paradigm, subjects either rest with eyes open fixating a fixation cross or with eyes closed. Usually, subjects are instructed not to fall asleep and to let their mind wander. Contrary to the simplicity of the paradigm, the generated database has a complex spatial structure and temporal dynamics, which arise from low-frequency fluctuations in the BOLD signal (Biswal et al., 1995; Fox and Raichle, 2007). Furthermore, the data are characterized by large number of spatial dimensions and they lack the temporal structure usually found for task-based fMRI investigations. Because of these two aspects, exploratory matrix factorization techniques are appropriate to analyze the large amount of data and to explore the complex spatial and temporal structure in the data, as shown by Kiviniemi et al. (2003). In this context, ICA-based pipelines have emerged as a state-of-the-art approach to investigate rs-fMRI data (Allen et al., 2011; Remes et al., 2011). This decomposition of resting-state fMRI data results in a so-called parcellation of the cortex into brain networks composed of functionally connected brain areas. In the literature, common brain networks are default-mode, cognitive control, visual, somatomotor, sub-cortical, auditory, or cerebellar, depending on the function of the brain areas included in each network (Allen et al., 2014), which can be successfully extracted from the data with ICA (Beckmann et al., 2005).

In this paper, a hybrid method is proposed for extracting resting-state networks (RSNs) from fMRI data based on constrained ICA (cICA) and empirical mode decomposition (EMD). This constrained extension of ICA optimizes the statistical independence and additionally the similarity to a given reference signal. In the framework of an augmented Lagrangian approach, the incorporation of a reference into ICA helps more robust ICs to be obtained while eliminating the ambiguities of the ICA approach (Lu and Rajapakse, 2006; Lin et al., 2009; Rodriguez et al., 2014). In this paper, like (Lin et al., 2009), spatial reference maps were employed to extract resting-state networks from fMRI data. Besides analyzing temporal time series (Huang et al., 1998), the EMD framework can be extended for the analysis of two-dimensional spatial maps (Nunes et al., 2003; Al-Baddai et al., 2016a,b), and in this study, we focus on the latter variant. Based on a preliminary study Wein et al. (2019), it is shown that an EMD-based image decomposition technique, denoted as Green's function in tension based bi-dimensional ensemble EMD (GiT-BEEMD) (Al-Baddai et al., 2016b), produces suitable references for cICA. This two-dimensional variant of EMD allows us to decompose images into so-called bi-dimensional intrinsic mode functions (BIMFs) and can also be used to slice-wise decompose volumetric fMRI images. Because of its inherent natural ordering of the extracted intrinsic modes according to their spatial frequencies, EMD can easily generate prototypical spatial maps. Similar spatial maps obtained with the EMD for each subject can be identified and averaged across subjects. In the next step, these prototypical spatial maps can serve as reference signals for a constrained ICA applied in parallel to the entire group of subjects. In this workflow, the references are obtained from the same dataset as used for the analysis, so no prior information is required. We extend previous constrained ICA methods (Lu and Rajapakse, 2006; Lin et al., 2009; Rodriguez et al., 2014) by showing that intrinsic mode functions generated by EMD are suitable references which help to extract resting-state networks in a purely data-driven fashion. The proposed workflow intrinsically adapts to the statistics of the given data, thereby avoiding any bias toward external references. We compare our new method to another data-driven ICA approach, an established group ICA method, based on temporal concatenation (Calhoun et al., 2001b), using a resting-state fMRI dataset from the *Human Connectome Project* (Essen et al., 2012). The potential benefits of this hybrid cICA-EMD method are emphasized by showing that this approach allows the user to actively shape the extracted resting-state networks. The trade-off between enforcing a certain similarity across subjects and preserving individual subject features can be determined and can be adapted to optimally fulfill the requirements of different studies.

## 2. Materials and Methods

The following subsections introduce the dataset employed and describe the data analysis techniques, which combine cICA and GiT-BEEMD, as well as the processing steps of the gICA approach used for comparison. Also, a flowchart of the proposed signal processing chain is provided.

### 2.1. Dataset

This study employed a data set from the *Human Connectome Project* (Essen et al., 2012). The S1200 release includes data from subjects who participated in four resting-state fMRI sessions, lasting 14.4 min each and resulting in 1200 volumes per session. Customized Siemens *Connectome Skyra* magnetic resonance imaging (MRI) scanners at Washington University with a field strength of B_0_ = 3 Tesla were employed for data acquisition, using a multi-band (factor 8) technique (Feinberg et al., 2010; Moeller et al., 2010; Setsompop et al., 2012; Xu et al., 2012). The data were collected by gradient-echo echo-planar imaging (EPI) sequences with a repetition time *TR* = 720*ms* and an echo time *TE* = 31.1 *ms*, using a flip angle of θ = 52°. The field of view was *FOV* = 208 *mm* × 180 *mm*, and *N*_*s*_ = 72 slices with a thickness of *d*_*s*_ = 2 *mm* were obtained, containing voxels with a size of 2 *mm* × 2 *mm* × 2 *mm*. The preprocessed version, which had been subjected to motion-correction, structural preprocessing, and ICA-FIX denoising, was chosen (Jenkinson et al., 2002, 2012; Fischl, 2012; Glasser et al., 2013; Smith et al., 2013; Griffanti et al., 2014; Salimi-Khorshidi et al., 2014). In the S1200 release, the FIX-classifier was trained on a labeled subset of the provided data (Griffanti et al., 2014; Salimi-Khorshidi et al., 2014). For comparison of the two approaches, 10 sessions from 10 different subjects were selected from the database. Gaussian smoothing with a half-width *FWHM* = 5 *mm* was then applied by using the *SPM12* software package^1^, and the first five images were discarded to account for magnetic saturation effects.

### 2.2. A Hybrid cICA-EMD Approach

**Figure 1:**
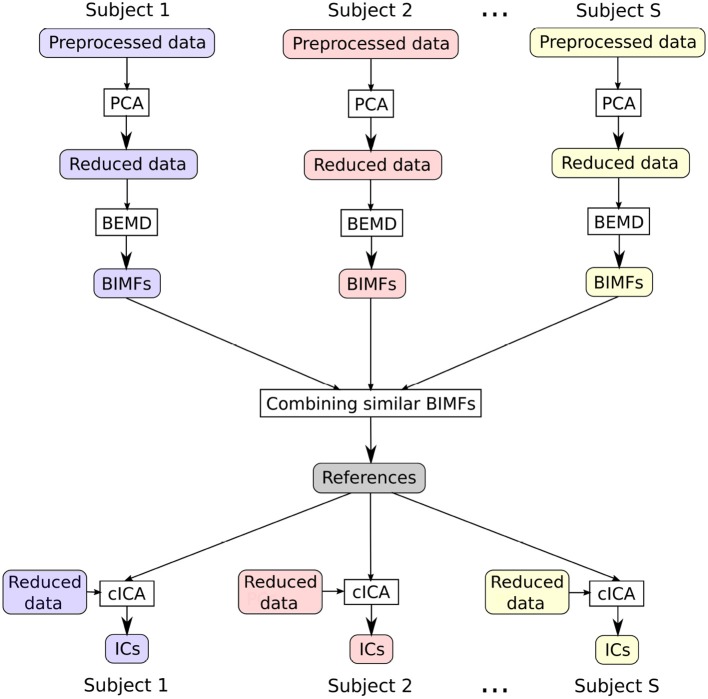
The flowchart sketches the main steps of the presented approach: first, reducing the data with PCA, then extracting BIMFs with spatial BEMD from the reduced data, and then combining the BIMFs of each subject in order to get shared references for cICA, which finally help to obtain comparable ICs across subjects.

In this section a new approach to deal with an ICA analysis across a group of subjects will be described. The flowchart in Figure 1 presents an overview of the various steps of the data analysis. All processing steps were performed in *MATLAB 9.3 Release 2017b*.

#### 2.2.1. Preprocessing

The data as obtained from the data repository will be further pre-processed, as explained in the following.

- In the first step, the voxel time series are linearly detrended and the voxel values are transformed to have zero mean and unit variance.
- Next a mask common to all subjects is used to exclude voxels that are located outside of the brain. The mask is created by employing the *GIFT* toolbox^2^, using the “Generate mask” option.
- The data of subject *s* is stored in a *K* × *L*-dimensional matrix **X**^(*s*)^ containing in its columns **x**_*l*_(*k*) the temporal evolution of *L* brain voxels at *K* time points.
- Principal component analysis (PCA) related dimension reduction is then performed based on the singular value decomposition (SVD) of **X**^(*s*)^ = **U**^(*s*)^**Σ**^(*s*)^**V**^(*s*)*T*^. If the row mean of **X**^(*s*)^ is removed, then **U**^(*s*)^ contains the eigenvectors of the covariance matrix **C**^(*s*)^ ∝ **X**^(*s*)^**X**^(*s*)*T*^ in its columns. The eigenvectors with the largest eigenvalues indicate the directions of greatest variance, which are denoted as principal components (PCs) (Jolliffe, 2014).
- Next, the fMRI images of each subject are projected onto the first *M* = 20 PCs ${\text{X}}_{M}^{\left(s\right)}={\left({\text{U}}_{M}^{\left(s\right)}\right)}^{T}{\text{X}}^{\left(s\right)}$. This reduces the number of images per session to *M* = 20 < *K*.
- Finally, from the reduced data sets, the image slices are reconstructed to enter into the GiT-BEEMD analysis, while the reduced data ${\text{X}}_{M}^{\left(s\right)}$ enters directly into the cICA processing path. The number of selected principal components *M* determines the number of sources, estimated in the cICA step. A relatively low order of *M* = 20 was chosen, to obtain robustly observed, large-scale resting-state networks (van den Heuvel and Pol, 2010) and to make it easy to identify extracted networks, which are suitable for comparison of results in section 3.

#### 2.2.2. Green's Function-Based Ensemble Empirical Mode Decomposition

For the next step, the extraction of suitable reference signals for cICA, the GiT-BEEMD technique was employed (Al-Baddai et al., 2016b) to extract intrinsic activity patterns from spatial volumetric fMRI images. The idea of this technique is to decompose a two-dimensional brain slice *I*(**r**) = *I*(*x, y*) into bi-dimensional intrinsic mode functions (BIMFs):

(1) $$\begin{array}{r} I\left(x,y\right)=\overset{J}{\underset{j=1}{\sum }}b_{j}\left(x,y\right) \end{array}$$

Here *b*_*j*_(*x, y*) denotes the *j*-th BIMF, which is estimated iteratively as described in Appendix 1, and, in our notation, we include the residuum *r*(*x, y*) as intrinsic mode *b*_*J*_(*x, y*). The first extracted BIMF contains the highest spatial frequency, which will decrease in every additionally extracted BIMF (Al-Baddai et al., 2016b).

**Figure 2:**
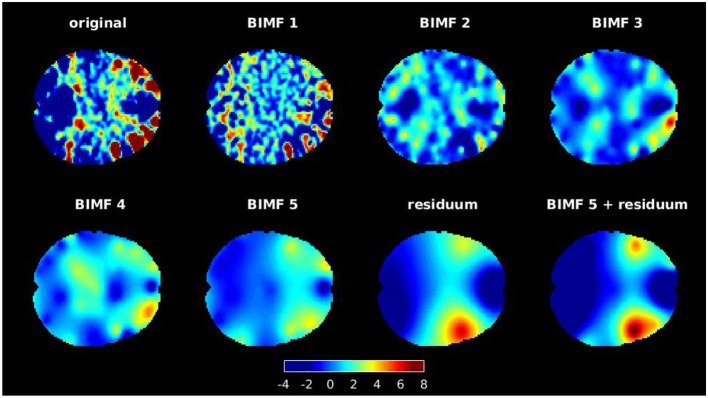
An example of decomposing a brain slice with GiT-BEEMD in the transverse anatomical plane. The slice was decomposed into five intrinsic modes and one residuum. Note that BIMF 1 contains the highest spatial frequencies, which gradually decrease for the other extracted BIMFs. Also, the combination of the fifth BIMF and residuum is illustrated, as this combination is used for further evaluations. A mask was applied after the decomposition to set all intensity values that were located outside of the scanned brain to zero.

Each brain slice was decomposed into five intrinsic modes and one residuum by repeating the sifting step five times. The ensemble step was repeated only twice, whereby noise was either added or subtracted from the data once at each step. The assisting noise was generated with a noise amplitude of *a*_η_ = 0.2. The tension parameter was initialized to *T*_1_ = 0.9 and reduced after the extraction of the *j*-th BIMF *b*_*j*_ to $T_{j+1}=T_{j}-\frac{1}{J}$. This avoids blob-like artifacts in low frequency modes if the tension parameter is set too high (Al-Baddai et al., 2016b). An example of a decomposition is provided in Figure 2. These intrinsic modes represent characteristic spatial textures of the activity distribution in the brain. BIMFs with high spatial frequencies (see BIMF 1 and BIMF 2, for example) show highly localized spatial activation patterns that, however, are spread all over the brain slice, while BIMFs with lower spatial frequencies (see BIMF 4 and BIMF 5, for example) concentrate the activity in few highly localized areas in the brain. In this example, the residuum reveals focused activity in the temporal brain network. Note that this reflects the high activity in this area seen in the original activity distribution of the chosen brain slice. A combination of lowest spatial frequency intrinsic mode plus the residuum, i. e., *b*_5_(*x, y*) + *b*_6_(*x, y*) ≡ *b*_56_(*x, y*), was used as reference for cICA in the further evaluations. The analysis in section 3 reveals that low-frequency modes are especially suitable to serve as references in order to obtain consistent resting-state networks across subjects. By decomposing the activity patterns and pointing toward specific brain areas, these intrinsic modes can help the ICA to converge to these specific areas for all subjects. Figure 4 shows that, for the most demanding similarity constraint, the most consistent results were achieved by combining BIMF 5 and the residuum to reference networks. This combination is also depicted in Figure 2. From all the decomposed two-dimensional slices, the corresponding modes *b*_56_(*x, y*) were organized into a three-dimensional data array, which then was concatenated into a 3D volume intrinsic mode function (VIMF). Decomposing the *M* = 20 brain volumes per subject in PC subspace results in *M* VIMFs per subject. For the next processing steps, the voxels inside of the brain are sorted into an *M* × *L* matrix again, denoted as ${\text{V}}_{M}^{\left(s\right)}$.

In order to extract from each subject RSNs that are consistent across the proband cohort, corresponding intrinsic modes need to be identified. As part of the minimal preprocessing pipeline of the *Human Connectome Project* (Glasser et al., 2013), the fully preprocessed fMRI data were transformed to the MNI standard space for every subject, and therefore a good correspondence of activity patterns between the healthy subjects was given. Note that if two different subjects cohorts were to be analyzed, the reference networks should be formed individually for each group, because in patients with neurological diseases, resting-state activity could fundamentally differ from that of healthy controls. In our work, studying only healthy probands, averaging most similar modes between subjects yields proper common reference signals for all subjects that have been employed in a cICA of fMRI datasets. A visual inspection of the VIMFs of any two randomly chosen subjects showed that corresponding spatial patterns might occur in different rows of ${\text{V}}_{M}^{\left(s\right)}$. Hence, the extracted VIMFs first need to be ordered according to their similarity between subjects. An efficient way to assign similar VIMFs between subjects is offered by the assignment algorithm proposed by Munkres (1957) based on the Hungarian method developed by Kuhn (1955). After computing the proper correspondences between the VIMFs of all subjects, the references **R** to be used in the cICA algorithm are then obtained by averaging the corresponding VIMFs across all subjects. To summarize, the reference signals are computed as follows:

1. Initialize the reference as $\text{R}={\text{V}}_{M}^{\left(1\right)}$
2. For *s* = 2, …, *S*, do:
  - Apply the Hungarian algorithm and re-order the rows of ${\text{V}}_{M}^{\left(s\right)}\to {\tilde{\text{V}}}_{M}^{\left(s\right)}$
  - Update the reference $\text{R}\leftarrow \frac{s-1}{s}\text{R}+\frac{1}{s}{\tilde{\text{V}}}_{M}^{\left(s\right)}$

To apply the Hungarian algorithm, a cost function $1-\rho \left(\text{R},{\text{V}}_{M}^{\left(s\right)}\right)$ is defined that, if optimized, results in an ordering of the rows in ${\text{V}}_{M}^{\left(s\right)}$ such that the sum of the correlation coefficients between pairs of rows in the two matrices is maximized. Note that the algorithm achieves the re-ordering without calculating all *M* possible assignments. Finally, each row of **R** is normalized, having entries with zero mean and unit variance, and *M* = 20 references for the cICA algorithm are obtained.

#### 2.2.3. Constrained ICA

Sources ${\text{Y}}_{M}=\left[{\text{y}}_{1},\ldots ,{\text{y}}_{L}\right],\text{y}\in {\mathbb{R}}^{M}$ can be blindly estimated from the mixtures ${\text{X}}_{M}=\left[{\text{x}}_{1},\ldots ,{\text{x}}_{L}\right],\text{x}\in {\mathbb{R}}^{M}$ according to:

(2) $$\begin{array}{l} {\text{Y}}_{M}=\text{W}{\text{X}}_{M} \end{array}$$

with the demixing matrix defined as $\text{W}={\left[{\text{w}}_{1},\ldots ,{\text{w}}_{M}\right]}^{T}$, where ${\text{w}}_{m}^{T}$ are the rows of the demixing matrix and **X**_*M*_ collects all *L* samples of the projected data after being spatially transformed to zero mean and unit variance.

Finding a demixing matrix is solved by designing an optimization problem where inequality and equality constraints are integrated in an augmented Lagrangian formulation. The inequality constraint terms in the Lagrange function are re-written as equality constraints with the help of a slack variable (Lu and Rajapakse, 2006). After finding the optimal value of these slack variables, the modified version of the augmented Lagrangian function is written as

(3) $$\begin{array}{l} L\left(\text{W},\mu \right)=J\left(\text{W}\right)\\ \;+\overset{M}{\underset{m=1}{\sum }}\frac{1}{2\gamma_{m}}\;\left[{\left(\max\left\{0,\gamma_{m}h_{m}\left({\text{w}}_{m}^{T}\right)+\mu_{m}\right\}\right)}^{2}-\mu_{m}^{2}\right] \end{array}$$

where μ_*m*_ are the Lagrangian parameters, while γ_*m*_ represents a user-defined penalty. The first term *J*(**W**) reflects the cost function of ICA, and the second term in the Lagrangian is related with the inequality constraint, which compares the *m*-th extracted component with the corresponding reference signal:

(4) $$\begin{array}{r} h\left({\text{w}}_{m}^{T}\right)=\varsigma_{m}-\epsilon \left({\text{w}}_{m}^{T}\text{x},r_{m}\right)\leq 0 \end{array}$$

where ϵ(·) is a similarity measure and ς_*m*_ is a threshold parameter. Similarity is conventionally expressed either through a correlation measure 𝔼{*y*_*m*_*r*_*m*_} or the mean squared error $𝔼\left\{{\left(y_{m}-r_{m}\right)}^{2}\right\}$, with $y_{m}={\text{w}}_{m}^{T}\text{x}$. The expected value is approximated by an average over the available data.

Estimating the demixing matrix **W**, given the constraint introduced above, can be achieved in different ways, based either on negentropy-like cost functions (points 1–3) or on a maximum likelihood estimate (point 4):

1. Simply one IC, most similar to the given reference signal, can be extracted. This approach can easily be extended to a multi-reference cICA. However, this additionally requires a decorrelation of the weights during each iteration to prevent different weights from converging to identical estimations (Lu and Rajapakse, 2006).
2. Lu and Rajapakse (2006) introduced an objective function for cICA that contained an additional equality constraint to bound the weights. Later, a simplification was introduced by Lin et al. (2007) where equality constraints were omitted; rather, the weight vectors were normalized at each iteration instead.
3. Also, cICA based on fixpoint learning (Lin et al., 2009) was proposed, which should overcome the limitations of the second-order Newton-like learning used in the cICA algorithm of Lu and Rajapakse (2006).
4. Finally, yet another version, using a cost function *J*(**W**) based on a maximum likelihood estimate has been proposed (Rodriguez et al., 2014) according to
  (5) $$\begin{array}{r} J\left(\text{W}\right)\approx 𝔼\left\{\overset{M}{\underset{m=1}{\sum }}\log\left(p\left({\text{w}}_{m}^{T}\text{x}\right)\right)\right\}+\log|\det\left(\text{W}\right)| \end{array}$$
  An iterative procedure is then derived to update the parameters $\mu ={\left[\mu_{1},\ldots ,\mu_{M}\right]}^{T}$ and the de-mixing matrix **W**. Thereby, a decoupling scheme based on a Gram-Schmidt orthogonalization is proposed, finally yielding the following objective function:
  (6) $$\begin{array}{r} J\left({\text{w}}_{m}\right)\propto 𝔼\left\{\log\left(p\left({\text{w}}_{m}^{T}\text{x}\right)\right)\right\}+\log|{\text{d}}_{m}^{T}{\text{w}}_{m}| \end{array}$$
  where the decoupling vector ${\text{d}}_{m}\in {\mathbb{R}}^{M\times 1}$ is defined through ${\tilde{\text{W}}}_{m}{\text{d}}_{m}=0$ and where ${\tilde{\text{W}}}_{m}\in {\mathbb{R}}^{\left(M-1\right)\times M}$ denotes the de-mixing matrix without entries to the *m*-th row.

It has been shown by Cardoso (1997) that a maximum likelihood approach to ICA is equivalent to the seminal Infomax approach put forward by Bell and Sejnowski (1995). Thus, in analogy to the maximum likelihood approach, a constrained and decoupled version of the extended Infomax algorithm can be obtained (Rodriguez et al., 2014). The extended Infomax algorithm is often used in an analysis of fMRI data (Correa et al., 2007) and was also used in this study as the basis of the cICA. A more detailed description of this algorithm and a proper metacode are given in Appendix 2 for the convenience of the reader.

The data, projected onto the first *M* = 20 PCs, and the *M* references, transformed to zero mean and unit variance, together enter the cICA algorithm to finally extract *M* = 20 ICs. The weights are initialized with small random values, and the learning rate for the weights is set to η = 0.5. The scalar penalty can be set to 3 (Rodriguez et al., 2014). The influence of the references can be well determined by adjusting the threshold parameter. Therefore different settings have been studied using the correlations 𝔼{*y*_*m*_*r*_*m*_} as distance measures, and the results are presented in section 3.

### 2.3. Group ICA

Generally, fMRI data are compared across a group of subjects by employing the gICA algorithm put forward by Calhoun and his group (Calhoun et al., 2001a). This gICA is made available in the *GIFT* toolbox^3^ and was incorporated in this study for comparison. Voxel time series were preprocessed by variance normalization through linearly detrending and transforming the data to zero mean and unit variance. The single-subject data matrices ${\text{X}}_{K\times L}^{\left(s\right)}$ enter the first PCA step, with the temporal evolution of *L* brain voxels at *K* time points in columns. The subject datasets were then projected onto the first *M*′ = 1.5 · *M* = 30 PCs in this step by applying an SVD to the data matrix. This follows the recommendation of the GIFT toolbox, projecting the data onto 1.5 times the components used in the group reduction step. Note that in the latter step, the number of projections was chosen to be *M* = 20. The *S* reduced *M*′ × *L* matrices ${\text{X}}_{M^{\prime }}^{\left(s\right)}$ on subject level were concatenated to an (*S* · *M*′) × *L* group matrix ${\text{X}}_{S\cdot M^{\prime }}^{\left(g\right)}$ entering the second PCA step. The group matrix is projected onto *M* = 20 PCs, resulting in a reduced *M* × *L* matrix ${\text{X}}_{M}^{\left(g\right)}$. The 20 group spatial maps **S**_*M*_ are extracted from ${\text{X}}_{M}^{\left(g\right)}=\text{A}{\text{S}}_{M}$ by the extended Infomax algorithm (Lee et al., 1999) and by additionally employing the ICASSO option (Himberg et al., 2004), running the ICA algorithm ten times with different initializations to assure greater stability. Finally *M* = 20 subject-specific spatial ${\text{S}}_{M}^{\left(s\right)}$ maps were obtained by the GICA3 (Erhardt et al., 2011) back-reconstruction approach. We compared these 20 brain networks, with the 20 networks we obtained from our approach. For visualization purposes, *M* mean networks 〈**S**_m*_〉, *m* = 1, …, *M* were obtained by averaging ${\text{S}}_{M}^{\left(s\right)}$ over the subjects, i. e., $\langle {\text{S}}_{M}\rangle =\frac{1}{S}\overset{S}{\underset{s=1}{\sum }}{\text{S}}_{M}^{\left(s\right)}$.

## 3. Results

**Figure 3:**
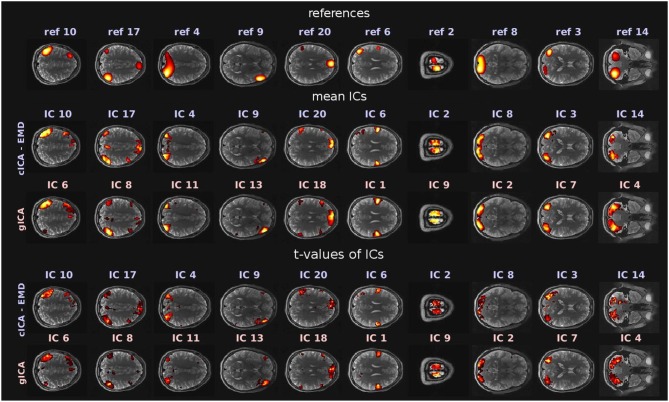
RSNs obtained by the two different approaches. The first row shows the references used for cICA, while the second row exhibits mean ICs, averaged over the subject cohort and computed with the newly proposed cICA-EMD method. These ICs are contrasted in the third row with mean ICs obtained by gICA. In the fourth and fifth row of this figure, *t*-values of the RSNs can be found. All depicted slices were chosen such that they intersect the peak activation voxel of the corresponding ICs obtained by gICA. For visualization purposes, the activations of the networks shown in the first three rows are normalized to zero mean and unit variance. Furthermore, in accordance with common usage, the voxel intensities Î(**r**) were thresholded by Î(**r**) > 2, and in the sixth and ninth column, the threshold was adjusted to Î(**r**) > 1.5 for better recognizability of the networks. The color range of the heatmap was adjusted to the largest intensity value in every pictured slice.

The goal of the study was to compare RSNs obtained with the newly proposed cICA-EMD approach as opposed to RSNs resulting from the conventional gICA approach. RSNs denote functionally connected brain areas that, however, are anatomically separated but maintain a high level of activity in a resting state of the proband. They are represented in this study by the ICs extracted with the discussed techniques. In this study, *M* = 20 ICs were extracted with either method. Comparable RSNs obtained by the different approaches were identified by visual inspection and are depicted in Figure 3. There, references used for cICA are shown in the first row, while in the second row, the ICs obtained therewith are presented, computed as mean ICs over subjects. In the third row of Figure 3, the mean ICs obtained by gICA are exhibited. The significance of the resulting ICs was tested with a one-sample student's *T*-test by employing the *SPM12* software package^4^. The resulting spatial maps of *t*-values are depicted in the fourth and fifth row in Figure 3. Spatial maps were thresholded at a significance level of *p* < 0.001 (*t* = 4.30, *df* = 9).

Most prominent brain areas are in IC 10/6 (obtained by cICA-EMD/gICA), the left inferior parietal lobule, in IC 17/8, the right angular/supramarginal gyrus, in IC 4/11, the superior occipital gyrus, in IC 9/13, the right inferior frontal gyrus, in IC 20/18, the anterior cingulate cortex, in IC 6/1, the precentral gyrus, in IC 2/9, the paracentral lobule, in IC 8/2, the middle occipital gyrus, and in IC 3/7, the middle temporal gyrus. The independent networks obtained represent well-observed RSNs (van den Heuvel and Pol, 2010) and can be further grouped based on their functions. The corresponding attentional and default mode networks are depicted in the first five columns, while the extracted auditory and sensorimotor networks are shown in the sixth and seventh column. Next, in the eighth and ninth column, visual networks are represented, and, in the last column, the cerebellum is shown. The similarity threshold for cICA was set to ς = 0.5 in this example. All resting-state networks obtained with both approaches, including the employed references, are provided in the Supplementary Material.

The motivation of different group ICA approaches is to make this explorative analysis technique suitable for studies where it is necessary to compare extracted networks between different subjects. This means that issues with permutation indeterminacy and reproducibility of ICA have to be overcome to obtain well-comparable networks. Therefore, a measure of interest for the evaluation of the two different approaches could be the consistency of activation patterns across subjects. This consistency was quantified by measuring how much a resting-state network ${\left({\text{y}}^{\left(s\right)}\right)}_{m}^{T}\in {\mathbb{R}}^{L}$ from one subject *s* differs on average from the mean network ${\langle \text{y}\rangle }_{m}^{T}=\frac{1}{S}\overset{S}{\underset{s=1}{\sum }}{\left({\text{y}}^{\left(s\right)}\right)}_{m}^{T}$ across subjects. Pearson's correlation $\rho {\left({\text{y}}^{\left(s\right)}\right)}_{m}^{T},{\langle \text{y}\rangle }_{m}$ was used to measure the correlation between standardized subject networks ${\text{y}}_{m}^{\left(s\right)}$ and the related mean networks ${\langle \text{y}\rangle }_{m}^{T}$. The following consistency measure is used:

(7) $$\begin{array}{r} K_{per}\left({\text{y}}_{m}\right)=\frac{1}{S}\overset{S}{\underset{s=1}{\sum }}\rho {\left({\text{y}}^{\left(s\right)}\right)}_{m}^{T},{\langle \text{y}\rangle }_{m} \end{array}$$

In order to find the most suitable references, the consistency measure was evaluated for different BIMFs or combinations of them. Following the process described in section 2.2.2, the GiT-BEEMD algorithm was used to decompose the activity patterns of the fMRI data into BIMFs, reflecting intrinsic patterns on different frequency scales.

**Figure 4:**
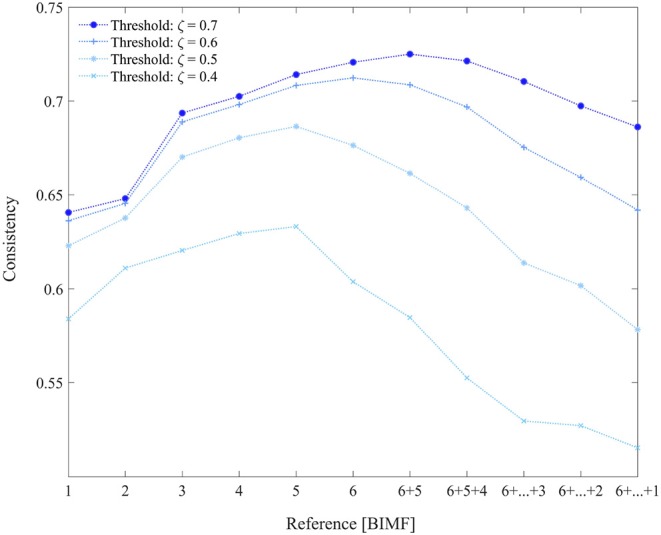
The consistency values of obtained resting-state networks with constrained ICA depending on the different BIMFs used as references. Also BIMFs were gradually summed up again, and combinations like BIMF 6 + 5 up to 6 + 5 + … + 1 were evaluated as potential reference signals.

Figure 4 depicts the consistency measure, as defined in Equation 7, with BIMF 1 to 6 used as references and its dependence on similarity threshold ζ. Extracted BIMFs were also gradually summed up again, and for the most demanding threshold, ζ = 0.7, a combination of BIMF 5 + BIMF 6 (the residuum) yielded the best results. This combination was used for further evaluations of our approach, and for the comparison with gICA (Calhoun et al., 2001b). For the comparison, the consistency measure was computed for all ICs obtained with the cICA-EMD approach and again for different settings of the similarity threshold ς. An equivalent procedure was followed using the results from applying the gICA algorithm. The results are listed in Table 1.

**Table 1:** Consistency values of ICs obtained by the proposed cICA-EMD approach for different settings of the similarity threshold ς, as well as the values of ICs obtained by gICA.

**cICA-EMD**										
**Threshold**	**IC #**									
	**10**	**17**	**4**	**9**	**20**	**6**	**2**	**8**	**3**	**14**
0.40	0.62	0.58	0.63	0.59	0.55	0.56	0.60	0.54	0.60	0.53
0.50	0.67	0.68	0.68	0.68	0.66	0.66	0.69	0.65	0.70	0.59
0.60	0.71	0.76	0.73	0.74	0.71	0.72	0.71	0.73	0.73	0.62
0.70	0.71	0.79	0.75	0.76	0.71	0.73	0.72	0.76	0.76	0.62
**gICA**										
	**IC #**									
	**6**	**8**	**11**	**13**	**18**	**1**	**9**	**2**	**7**	**4**
	0.73	0.65	0.68	0.70	0.66	0.66	0.66	0.70	0.71	0.60

*ICs are sorted as in Figure 3 so that each column shows consistency values of comparable extracted networks*.

**Figure 5:**
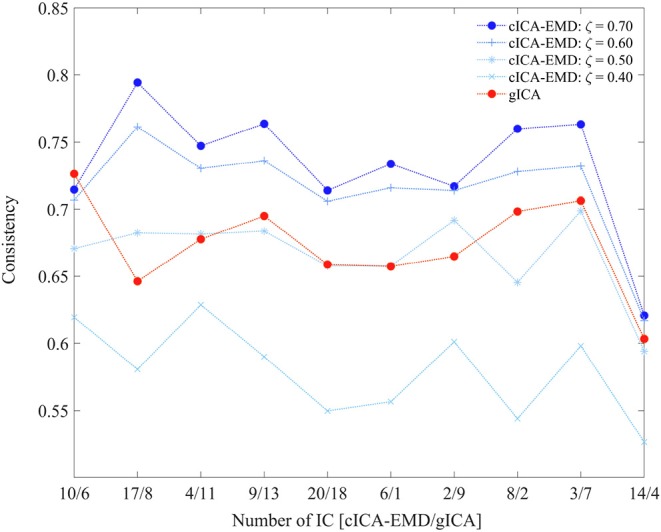
Consistency values of the respective ICs. The numbers on the x-axis refer to the IC number of the cICA-EMD vs. the gICA approach.

By adjusting the threshold parameter ς, it is possible to well determine the influence of the constraint during the optimization, so choosing a smaller threshold allows for more variability in the estimated components across subjects. Increasing the threshold increases the similarity between subject-specific components and common references. This means that if the similarity between every subject component and the shared reference increases, the similarity of components across subjects will also increase, which is quantified by the consistency measure in Equation 7. Figure 5 illustrates the behavior of the consistency at different similarity thresholds ς in comparison to gICA. If the threshold parameter is set to a value of ς = 0.40, the consistency is lower than that of gICA. By further increasing this threshold to a value of ς = 0.60, the consistency of estimated resting-state networks with the proposed approach starts to exceed that of gICA.

## 4. Discussion

The motivation of this paper was to propose a novel workflow for extracting resting-state networks that are consistent across a group of subjects. First, the dimensionality of the fMRI dataset was reduced at subject level with PCA. Intrinsic modes were extracted from the data by employing the GiT-BEEMD algorithm (Al-Baddai et al., 2016b). These subject-specific intrinsic modes reflect spatial activity patterns at different spatial frequencies. Hence, for each underlying spatial frequency, a common reference mode can be formed. It turned out that low-frequency modes concentrated the activity into spatially contiguous patterns and were especially well-suited to serve as reference modes for the extraction of independent components with a cICA algorithm. Note that if intrinsic spatial modes, which are naturally ordered according to their dominant local spatial frequency, are chosen as reference signals within a cICA, the resulting independent modes are also ordered in correspondence to their assigned intrinsic modes. Thus, the natural ordering of the intrinsic modes with respect to their spatial frequencies helps to overcome the permutation ambiguity of ICA in extracting consistent resting-state networks across subjects. Previously, references for constrained ICA had to be predefined, in the form of temporal activation profiles or anatomical regions of interest derived from an atlas (Lu and Rajapakse, 2006; Lin et al., 2009; Rodriguez et al., 2014). In the absence of any stimulus, like in resting-state fMRI, such temporal profiles might not be available. Also, in the case of patients with neurological disorders, spatial priors can be inappropriate. Atlases are typically defined based on a cohort of healthy subjects, meaning that reference brain networks defined by the latter might unduly bias the outcome of the analysis. Therefore it was our goal to establish a purely data-driven workflow by hybridizing cICA with EMD and obtaining references from the same data as used in the study. We demonstrated that our fMRI data processing pipeline produces commonly observed resting-state patterns. These functional networks were then compared to those obtained by the widely used gICA, which is based on a temporal concatenation of individual datasets (Calhoun et al., 2001b). It was shown that with the constrained extended Infomax algorithm (Rodriguez et al., 2014), the influence of the references upon the estimation of the related ICs could be controlled well. Based on the mathematically well-described augmented Lagrangian framework in our workflow, it is transparent to the user with respect to how homologous resting-state networks across subjects are deduced. In the processing pipeline presented, the cICA-EMD approach also allowed the optimization procedure to be shaped by adjusting the threshold parameter, which determines the impact of the reference on the IC extraction. By choosing a lower threshold, e.g., allowing for a lower similarity to the references, more freedom could be given to the exploratory character of ICA and the formation of subject-specific features. Defining a high threshold resulted, across subjects, in a higher consistency of the extracted resting-state networks. These RSNs were even more consistent than those obtained by the conventional gICA. Although there is no exact ground truth on how resting-state networks should ideally look, the threshold can be chosen in a way such that the obtained networks optimally fulfill the requirements of a particular study. For example, when performing a classification task, the threshold can be chosen to maximize the accuracy of the classifier. Thus, the good interpretability and high flexibility of the proposed processing pipeline can offer beneficial properties for application in resting-state studies. Besides applications of EMD for time series analysis in functional MRI (Qian et al., 2015; Goldhacker et al., 2018; Zhang et al., 2018), we showed that spatial EMD can also be used to extract useful intrinsic patterns from functional MRI data, representing characteristic resting-state activations. This could further motivate other researchers to consider the spatial variant of this technique and to investigate other applications of this method in the field of MRI.

## Data Availability Statement

Publicly available datasets were analyzed in this study. This data can be found here: https://db.humanconnectome.org/.

## Author Contributions

SW and EL designed the study. SW and MG performed the simulations. SW, EL, AT, and MG wrote the manuscript. MWG and EL supervised the study and revised earlier versions of this manuscript.

### Conflict of Interest

The authors declare that the research was conducted in the absence of any commercial or financial relationships that could be construed as a potential conflict of interest.

## Footnotes

^1^https://www.fil.ion.ucl.ac.uk/spm/software/spm12/

^2^http://mialab.mrn.org/software/gift/

^3^http://mialab.mrn.org/software/gift/

^4^https://www.fil.ion.ucl.ac.uk/spm/software/spm12/
