# Supplementary material for: A Constrained ICA-EMD Model for Group Level fMRI Analysis
Source: Front Neurosci. 2020 Apr 15;14:221. doi: 10.3389/fnins.2020.00221 (PMC7175031; doi:10.3389/fnins.2020.00221)
Supplement: Supplementary file 1 [file Data_Sheet_1.PDF]

Supplementary material

1. References for cICA

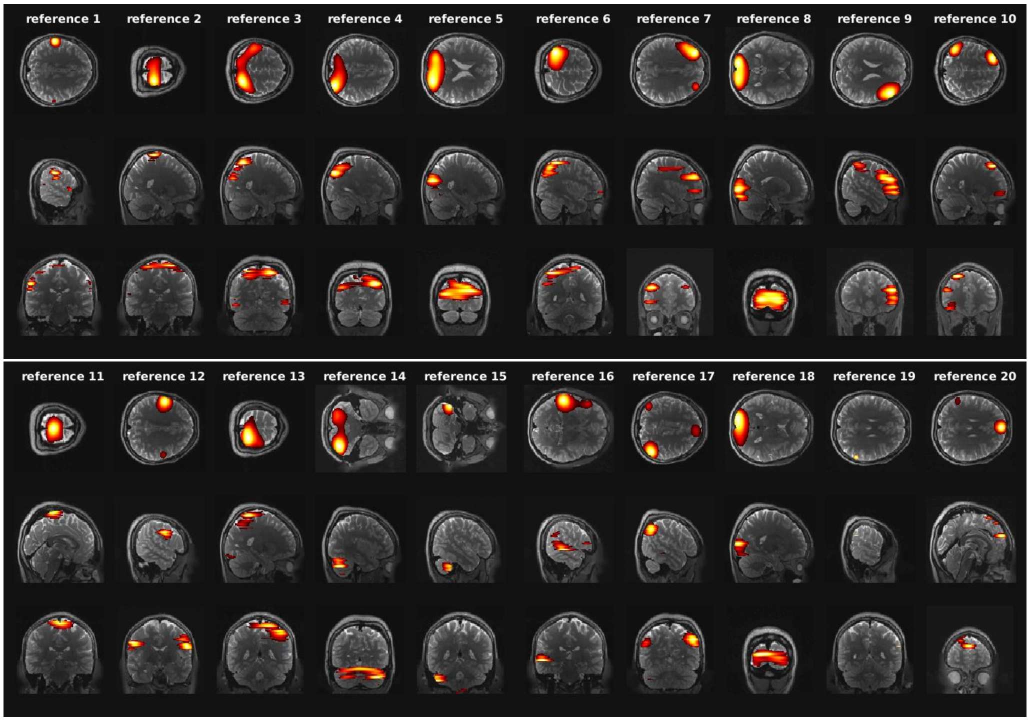

Figure 1: References used for cICA. The references are presented in the three principal anatomical planes: transverse, sagittal and frontal. The three planes intersect the voxel with highest intensity value in every volumetric image. For visualization purposes, the activations of the networks shown are normalized to zero mean and unit variance, and the intensity of the pictured brain slice $\hat{I}(\mathbf{r})$ was thresholded by $\hat{I}(\mathbf{r}) > 2$. The color range is adjusted to the largest intensity value in every pictured slice.

2. ICs obtained by the cICA - EMD approach

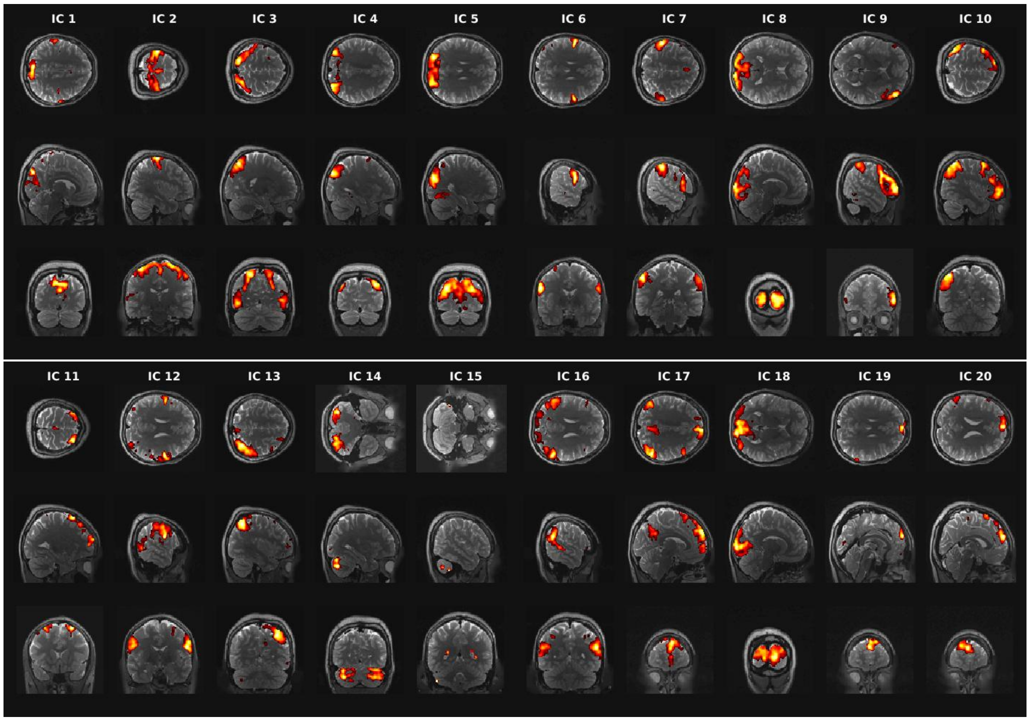

Figure 2: ICs obtained by the proposed approach. The depicted ICs are computed as mean ICs over subjects. Here the similarity threshold for cICA was set to $\varsigma_m = 0.5$. The ICs are presented in the three principal anatomical planes: transverse, sagittal and frontal. The three planes intersect the voxel with highest intensity value in every volumetric image. For visualization purposes, the activations of the networks shown are normalized to zero mean and unit variance, and the intensity of the pictured brain slice $\hat{I}(\mathbf{r})$ was thresholded by $\hat{I}(\mathbf{r}) > 2$. The color range is adjusted to the largest intensity value in every pictured slice.

3. ICs obtained by gICA

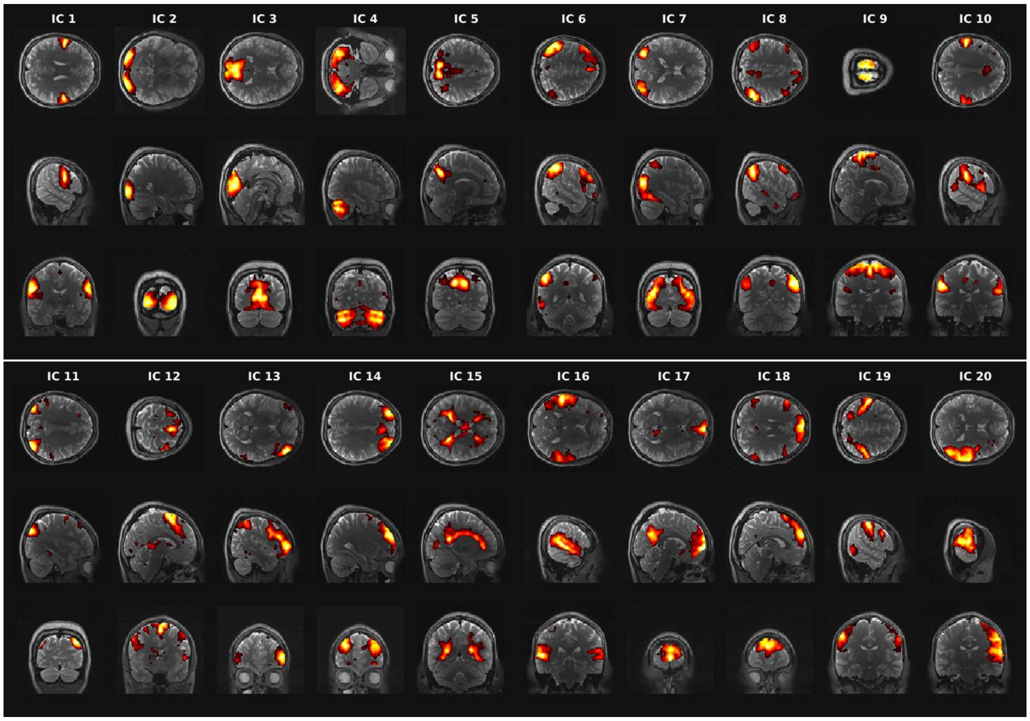

Figure 3: ICs obtained by gICA. The depicted ICs are computed as mean ICs over subjects. The ICs are presented in the three principal anatomical planes: transverse, sagittal and frontal. The three planes intersect the voxel with highest intensity value in every volumetric image. For visualization purposes, the activations of the networks shown are normalized to zero mean and unit variance, and the intensity of the pictured brain slice $\hat{I}(\mathbf{r})$ was thresholded by $\hat{I}(\mathbf{r}) > 2$. The color range is adjusted to the largest intensity value in every pictured slice.
