# Supplementary material for: A Constrained ICA-EMD Model for Group Level fMRI Analysis
Source: Front Neurosci. 2020 Apr 15;14:221. doi: 10.3389/fnins.2020.00221 (PMC7175031; doi:10.3389/fnins.2020.00221)
Supplement: Supplementary file 2 [file Data_Sheet_2.pdf]

1 A GREEN'S FUNCTION-BASED BI-DIMENSIONAL EMPIRICAL MODE DECOMPOSITION

Empirical mode decomposition (EMD) (Huang et al., 1998; Zeiler et al., 2011) enables to locally extract simple oscillatory components from any multi-variate signal, denoted as intrinsic mode functions (IMFs). The obtained spatial oscillations represent characteristic textures of the given data. The process generating these IMFs, is usually denoted as sifting, which results in pure oscillations with spatially and/or temporally varying amplitude and frequency. Note that in contrary to classical filtering methods, EMD is a purely data driven approach, which intrinsically adapts to the statistics of the data, and does not rely on a predefined frequency range. Also IMFs are naturally ordered according to their characteristic (spatial)-frequencies.

The one-dimensional EMD can be extended to an image decomposition technique, denoted as bi-dimensional EMD (BEMD). The process of extracting bi-dimensional IMFs (BIMFs) $b_j(x,y)$ is summarized in the following steps (Al-Baddai et al., 2016a; Nunes et al., 2003):

Bi-dimensional empirical mode decomposition

---

1. Choose the number of intrinsic modes J and the number of sifting steps N and set $r(x,y) = f(x,y)$
2. Extract the j-th BIMF by repeating the sifting steps N times:
   a. Identify all local maxima and minima of the array $r(x,y)$
   b. Interpolate these local maxima to an upper envelope surface $s_{max}(x,y)$ and local minima to a lower envelope surface $s_{min}(x,y)$ and calculate the mean between upper envelope surface and lower envelope surface $s_{mean}(x,y) = 0.5\,(s_{max}(x,y) + s_{min}(x,y))$
   c. Update $r(x,y)$ with $r(x,y) \leftarrow r(x,y) - s_{mean}(x,y)$
   d. If loop is finished, set $b_j(x,y) = r(x,y)$, otherwise repeat steps (a) - (d)
3. Subtract all calculated BIMFs $b_{1\ldots j}$ from $f(x,y)$ to obtain new $r(x,y) = f(x,y) - \sum_{j<j+1} b_j(x,y)$
4. If all J BIMFs are extracted, $r(x,y)$ is the residuum, otherwise repeat step 2 to compute the next BIMF b_{j+1}

Interpolation schemes, which are used to describe the upper and lower envelope surface, usually suffer from problems like *computational load*, *boundary artefacts* and *over- and undershooting* (Al-Baddai et al., 2016b). Using a Green's function-based interpolation scheme, local maxima or minima can be considered as the known points of the envelope surface, which can be found with an 8-connected neighborhood strategy. Then surface envelopes $s(\mathbf{r}_u)$, at Cartesian coordinates $\mathbf{r}_u = [x_u, y_u]^T$, are represented as a weighted sum of Green's functions (Wessel and Bercovici, 1998):

$$s(\mathbf{r}_u) = \sum_{n=1}^{N} v_n \Phi(\mathbf{r}_u, \mathbf{r}_n), \tag{1}$$

where $\Phi(\mathbf{r}_u, \mathbf{r}_n)$ represent the Green's functions and v_n the corresponding weights. Further $\mathbf{r}_u$ denotes a point where the surface is unknown and $\mathbf{r}_n$ describes the n-th constraint, which corresponds to a local extremum. The Green's function, expressed in 2D-Cartesian coordinates, reads as:

$$\Phi(\mathbf{r}_u, \mathbf{r}_n) = \log(p|\mathbf{r}_u - \mathbf{r}_n|) + K_0(p|\mathbf{r}_u - \mathbf{r}_n|) \tag{2}$$

with $K_0(\cdot)$ representing the modified Bessel function of the second kind and zero order and $|\cdot|$ the Euclidean distance. Here $p^2 = \frac{T}{D}$ is related with tension T at the boundaries, and D describes the flexural rigidity of the surface (Wessel and Bercovici, 1998). The estimation of the envelope surface is based on two steps. In a first step weights v_n can be estimated by taking the known values of local maxima (or minima) as the values $s(\mathbf{r}_n) = [s(\mathbf{r}_1), \ldots, s(\mathbf{r}_N)]^T$ in a total of N locations $\mathbf{r}_n$ and solving a linear system of N equations, described by equation 1. In a second step, if the weights v_n are now obtained, the surface can be estimated at any point $\mathbf{r}_u$.

To avoid mode mixing and boundary artifacts, a noise assisted ensemble version of the Green's function based BEMD (GiT-BEEMD) can be used (Al-Baddai et al., 2016a). Adding and subtracting noise η from the original image $f(x, y)$ leads to two noisy versions $\tilde{f}(x, y)^* : \tilde{f}^+(x, y) = f(x, y) + \eta$ and $\tilde{f}^-(x, y) = f(x, y) - \eta$. Both versions $\tilde{f}(x, y)^*$ can now be decomposed into BIMFs. By computing the mean as $0.5\,(\tilde{f}^+(x, y) + \tilde{f}^-(x, y))$ the original array $f(x, y)$ could be reconstructed and therefore after decomposing the two versions $\tilde{f}(x, y)^*$, the BIMFs of $f(x, y)$ can be naturally obtained by averaging BIMFs of the noisy versions. For this version of BEMD it is sufficient to use a few ensemble steps only to improve the image quality significantly (Al-Baddai et al., 2016b), reducing computational load. The two-dimensional image decomposition was applied to the slices of volumetric fMRI images in the transverse anatomical plane.

2 NON-ORTHOGONAL CONSTRAINED EXTENDED INFOMAX

The objective function for ICA, based on Maximum Likelihood, can be derived as (Hyvärinen et al., 2001)

$$J(\mathbf{W}) \approx \mathbb{E}\Big\{ \sum_{m=1}^{M} \log(p(\mathbf{w}_m^T \mathbf{x})) \Big\} + \log|\det(\mathbf{W})| \tag{3}$$

It is proposed a decoupling strategy for the second term of cost function resulting in a objective function for each of the rows of the mixing matrix (Rodriguez et al., 2014)

$$J(\mathbf{w}_m) \approx \mathbb{E}\left\{ \log(p(\mathbf{w}_m^T \mathbf{x})) \right\} + \log|(\mathbf{d}_m^T \mathbf{w}_m)| + \log(S) \tag{4}$$

with $S = \sqrt{|\det\left(\tilde{\mathbf{W}}_m \tilde{\mathbf{W}}_m^T\right)|}$, where $\tilde{\mathbf{W}}_m$ is the de-mixing matrix without $m-th$ row. The decoupling vector is the vector $\tilde{\mathbf{W}}_m \mathbf{d}_m = 0$ and can be computed as

$$\mathbf{d}_m = (\mathbf{I} - \tilde{\mathbf{W}}_m^T (\tilde{\mathbf{W}}_m \tilde{\mathbf{W}}_m^T)^{-1} \tilde{\mathbf{W}}_m)\mathbf{v} \tag{5}$$

where $\mathbf{v}$ is a vector with Gaussian random values. The vector gradient of this function is then derived as

$$\nabla_{\mathbf{w}_m} J(\mathbf{w}_m) = \mathbb{E}\left\{ f_m(\mathbf{w}_m^T \mathbf{x}) \mathbf{x}^T \right\} + \frac{\mathbf{d}_m^T}{\mathbf{d}_m^T \mathbf{w}_m} \tag{6}$$

with so-called score functions defined as

$$f_m(y_m) = \frac{\partial \log p(y_m)}{\partial y_m} \tag{7}$$

The extended Infomax algorithm defines these non-linearities $f_m(y_m)$ differently for sub-Gaussian or super-Gaussian components (Lee et al., 1999). The Lagrangian multipliers μ_m are updated by gradient ascent

$$\mu_m \leftarrow \max\{0, \gamma_m h_m(y_m, r_m) + \mu_m\} \tag{8}$$

For the algorithm a convergence metric like $[(vec(\Delta\mathbf{W}))^T vec(\Delta\mathbf{W})] < \tau$ can be adapted (Rodriguez et al., 2014). Here $\Delta\mathbf{W}$ is the element by element difference of $\mathbf{W}$ after each iteration, $vec(\cdot)$ stores all elements of a matrix in a column vector and τ is the tolerance value. So the non-orthogonal constrained extended Infomax can be summarized in the following steps:

Non-orthogonal constrained extended Infomax

---

1. Randomly initialize $\mathbf{W}$ and initialize μ_m, set γ_m and thresholds ς_m
2. **for** weights $\mathbf{w}_m,\ m = 1, \ldots, M$ do:
   a. Compute the vector $\mathbf{d}_m = (\mathbf{I} - \tilde{\mathbf{W}}_m^T(\tilde{\mathbf{W}}_m\tilde{\mathbf{W}}_m^T)^{-1}\tilde{\mathbf{W}}_m)\mathbf{v}$, with $\mathbf{d}_m \perp \mathbf{w}_{i\neq m}$. Here $\mathbf{v}$ is a Gaussian random vector.
   b. Compute $\mathbf{y}_m = \mathbf{w}_m^T\mathbf{x}$
   c. Update $\mu_m \leftarrow \max\{0, \gamma_m h_m(y_m, r_m) + \mu_m\}$
   d. Let $\Delta\mathbf{w}_m^T \propto \frac{\mathbf{d}_m^T}{\mathbf{d}_m^T\mathbf{w}_m} + \mathbb{E}\{f_m(\mathbf{w}_m^T\mathbf{x})\mathbf{x}^T\} - \frac{1}{2}\,\mu_m\mathbb{E}\{h'_m(y_m, r_m)\mathbf{x}^T\}$
      and set $f_m(y_m) = \tanh(y_m) - y_m$ for sub-Gaussian sources and
      $f_m(y_m) = -\tanh(y_m) - y_m$ for super-Gaussian sources.
   e. Update $\mathbf{w}_m \leftarrow \mathbf{w}_m + \Delta\mathbf{w}_m$
   f. And normalize $\mathbf{w}_m \leftarrow \frac{\mathbf{w}_m}{\|\mathbf{w}_m\|}$
   end for
3. Repeat step 2 until convergence.

REFERENCES

Al-Baddai, S., Al-Subari, K., Tomé, A. M., Ludwig, B., Salas-Gonzales, D., and Lang, E. W. (2016a). Analysis of fMRI images with bi-dimensional empirical mode decomposition based-on Green's functions. *Biomedical Signal Processing and Control* 30, 53 – 63. doi:10.1016/j.bspc.2016.06.019

Al-Baddai, S., Al-Subari, K., Tomé, A. M., Sol-Casals, J., and Lang, E. W. (2016b). A Green's function-based Bi-dimensional empirical mode decomposition. *Information Sciences* 348, 305 – 321. doi:10.1016/j.ins.2016.01.089

Huang, N., Shen, Z., Long, S., Wu, M., Shih, H., Zheng, Q., et al. (1998). The empirical mode decomposition and the hilbert spectrum for nonlinear and non-stationary time series analysis. *Proceedings of the Royal Society of London. Series A: Mathematical, Physical and Engineering Sciences* 454, 903–995

Hyvärinen, A., Karhunen, J., and Oja, E. (2001). *Independent Component Analysis*, vol. 26

Lee, T.-W., Girolami, M. A., and Sejnowski, T. J. (1999). Independent component analysis using an extended infomax algorithm for mixed sub-gaussian and super-gaussian sources. *Neural computation* 11 2, 417–41

Nunes, J. C., Bouaoune, Y., Deléchelle, É., Niang, O., and Bunel, P. (2003). Image analysis by bidimensional empirical mode decomposition. *Image Vision Comput.* 21, 1019–1026

Rodriguez, P. A., Anderson, M. Z., Li, X.-L., and Adalı, T. (2014). General non-orthogonal constrained ICA. *IEEE Transactions on Signal Processing* 62, 2778–2786

Wessel, P. and Bercovici, D. (1998). Interpolation with splines in tension: A Green's function approach 30, 77–93

Zeiler, A., Faltermeier, R., Böhm, M., Keck, I., Tomé, A., Puntonet, C., et al. (2011). *Empirical Mode Decomposition Techniques for Biomedical Time Series Analysis*. 60–81
